# Identifying Accessibility Requests for Patients With Disabilities Through an Electronic Health Record–Based Questionnaire

**DOI:** 10.1001/jamanetworkopen.2022.6555

**Published:** 2022-04-08

**Authors:** Varshini Varadaraj, Xinxing Guo, Nicholas S. Reed, Kerry Smith, Michael V. Boland, A. J. Nanayakkara, Bonnielin K. Swenor

**Affiliations:** 1 Wilmer Eye Institute, Johns Hopkins University School of Medicine, Baltimore, Maryland; 2 Johns Hopkins Disability Health Research Center, Baltimore, Maryland; 3 Department of Otolaryngology, Johns Hopkins University School of Medicine, Baltimore, Maryland; 4 Facebook, Global Accessibility Compliance, Washington, DC; 5 Massachusetts Eye and Ear and Harvard Medical School, Boston; 6 Johns Hopkins University School of Nursing, Baltimore, Maryland

## Abstract

**Importance:**

People with disabilities experience disparities in health care access and outcomes, and inaccessible health care facilities are major barriers to health care access. Methods to collect accessibility request information are needed to improve health care outcomes for patients with disabilities.

**Objective:**

To evaluate an electronic health record (EHR)–based questionnaire designed to identify accessibility requests for patients with disabilities at an eye clinic.

**Design, Setting, and Participants:**

This cross-sectional pilot study implemented an EHR questionnaire and prospectively collected data on accessibility requests at a university-based eye clinic. The study included 55 722 patients making appointments at the Johns Hopkins Wilmer Eye Institute from April 1, 2019, to March 31, 2020.

**Main Outcomes and Measures:**

The Wilmer Eye Institute staff were trained to assess accessibility requests of patients making appointments in-person or via telephone using a standardized script and entering patient responses into the EHR. Data were later extracted for analysis and used to determine the proportion of patients making eye appointments who reported a disability accessibility request (physical, sensory, or intellectual) during their clinic visit.

**Results:**

Accessibility request data were collected from 250 932 patient encounters. Patients had a mean (SD) age of 61.9 (20.6) years; most were women (146 846 [58.5%]) and were White individuals (162 720 [64.9%]). Of these, 23 510 (9.4%) encounters were associated with an accessibility request. The most reported accessibility request was mobility related (18 857 [7.5%]) (needing a cane, crutches, motorized scooter, walker, wheelchair, stretcher, assistance standing, or transport services), followed by sensory-related (2988 [1.2%]) (visual, hearing, or speech impairment), intellectual (353 [0.1%]), and other (1312 [0.5%]) (assistance with filling forms or service animal) requests. Patients with an accessibility request compared with those without, were older (72.6 vs 60.8 years), less likely to be White individuals (59.7% vs 65.4%), and more likely to be women (62.6% vs 58.1%), receiving Medicare (69.6% vs 41.5%), and have vision impairment (41.3% vs 13.6%) (*P* < .001 for all).

**Conclusions and Relevance:**

In this cross-sectional study, a substantial proportion of patients making eye appointments reported having accessibility requests as captured using a new EHR-based questionnaire. Such standardization of data collection for disability-related accessibility requests in EHR is scalable, could be expanded to other clinical settings, and has the potential to improve accessibility of health care interactions for patients with disabilities.

## Key Points

**Question:**

What proportion of patients presenting to an eye clinic have accessibility requests identified by implementing an electronic health record–based questionnaire?

**Findings:**

In this cross-sectional study evaluating 250 932 patient encounters at a university-based eye clinic, 9.4% of patients making eye care appointments reported having an accessibility request. The most commonly reported accessibility request was mobility related, followed by sensory-related (including visual) and intellectual-related requests.

**Meaning:**

Findings of this study suggest that this novel electronic health record–based questionnaire is scalable and can capture accessibility request information in a standardized manner across health care settings.

## Teaser

This cross-sectional study evaluates an electronic health record–based questionnaire designed to identify patient accessibility requests.

## Introduction

Sixty-one million community-dwelling US adults have a disability, representing 26% of the US population.^1^ As the US population ages, this number is set to increase substantially over time.^2,3^ Despite protections, mandates, and guidelines over the years from the Americans with Disabilities Act and beyond,^4,5,6^ people with disabilities continue to face considerable health inequities.^7,8,9,10^ They experience greater mortality and morbidity rates, often related to underscreening, underdiagnosis, and undermanagement of chronic health conditions.^11,12,13,14^ Therefore, with the aging US population, minimizing and ultimately eliminating health and health care disparities is of national importance.

Accessing health care remains a hurdle for people with disabilities.^2,15,16^ Categories of barriers include architecture, attitudes, and communication; for example, inadequate transport services for people with mobility limitations, lacking Braille or large-print materials for vision impairment, and sign language interpreters for hearing impairment.^2,16,17,18^ However, to provide services and accommodations that make hospitals and health care systems more accessible to people with disabilities, including vision impairment, we first need reliable estimates of the number of people reporting accessibility requests in health care settings. Currently, there are gaps in the collection of such data.

To begin to understand and improve the process of addressing the needs of people with disabilities, we developed a novel electronic health record (EHR)–based questionnaire to identify accessibility requests for patients with disabilities at a university-based eye clinic. In this article, we describe the questionnaire and estimates of accessibility requests in this setting.

## Methods

### Study Setting

This cross-sectional study was conducted at the Johns Hopkins Wilmer Eye Institute (including the main East Baltimore facility and all satellite clinics) from April 1, 2019, to March 31, 2020. A single EHR system (EpicCare Ambulatory, Epic Systems) was in place during this 1-year study period. The Johns Hopkins University School of Medicine Institutional Review Board reviewed and determined that this project qualifies as exempt research under US Department of Health and Human Services guidelines. For this reason, patient consent was not obtained. Race and ethnicity were self-reported by patients using several options provided. Race and ethnicity were assessed in this study to examine potential group differences in accessibility requests. We followed the Strengthening the Reporting of Observational Studies in Epidemiology (STROBE) guidelines for cross-sectional studies.^19^

### Survey Development and Implementation

The Americans with Disabilities Act and Accessibility Program Manager at Johns Hopkins Medicine and collaborating staff from the Johns Hopkins Epic team developed and implemented the questionnaire. Before this project, all patients making an appointment at the Wilmer Eye Institute were not consistently queried on their accessibility requests. When patients volunteered information on their accessibility requests, a free-text option was entered in their EHR to document any accessibility requests. With the latest set of patient EHR portal updates in April 2019, the workflow to collect accessibility requests data was converted from the free-text field to a discrete form allowing the more systematic collection of disability information. In this updated format, accessibility requests were collected in a special needs fields under “additional demographics,” which can be accessed during appointment scheduling (Figure). More than 1 accessibility request could be documented per patient, as needed. The first accessibility request reported was recorded as the primary accessibility request, and any additional requests were recorded in subsequent and additional special needs fields. Note this special needs field was not created or named by this project or study team, but instead this project used an existing EHR field, and the authors recognize this term is not preferred by many people with disabilities.^20,21^

**Figure.**
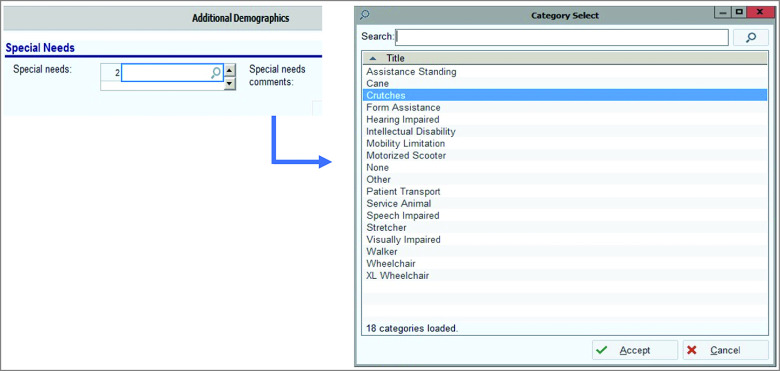
Accessibility Requests Collected Under the New Pilot Workflow at the Johns Hopkins Wilmer Eye Institute From April 2019 to March 2020

The Wilmer Eye Institute call center and front-desk staff were trained to assess accessibility requests of patients either calling in to make appointments or making appointments in person during checkout using a standard script (eAppendix in the Supplement), and patient responses were recorded in the EHR. Any patients who requested appointments electronically (ie, via the Johns Hopkins MyChart [Epic Systems], a web platform that connects patients to the health care team) did not encounter these accessibility request questions.

### Statistical Analysis

Records of all patients who provided information on their accessibility requests between April 1, 2019, and March 31, 2020, were included in these analyses. Demographic information (age, sex, race, ethnicity, and insurance) and vision data were also extracted from their EHR. Distance visual acuity data collected included presenting, pinhole (if available), and manifest refraction (if available). Vision impairment was defined as better-eye visual acuity worse than 20/40 based on the best-available acuity measure. The primary outcome variable was the proportion of patients making eye appointments who reported having any disability need and requested support for accessibility during their clinic visit. The proportion of patients who reported having an additional disability was also calculated.

We defined someone as having an accessibility request if they answered “Yes” to the main question: “Do you have any accessibility requests for this visit?” (eAppendix in the Supplement). Patients subsequently specified their accessibility request from a list of 18 options (including an “other” option) (Figure). The 18 types of accessibility requests that were reported were categorized as follows:

1. Mobility: cane, crutches, motorized scooter, walker, wheelchair, stretcher, assistance standing, or transport services
2. Sensory: assistance due to visual, hearing, or speech impairment
3. Intellectual: assistance due to an intellectual/developmental disability
4. Other: requiring assistance with filling forms, use of a service animal, or other reasons

Sociodemographic characteristics were summarized across groups with and without any accessibility requests using Pearson χ^2^ and *t* tests for continuous and categorical data, respectively. The proportions of patient encounters with missing data on accessibility requests were calculated for each quarter of the study period (April to June 2019, July to September 2019, October to December 2019, and January to March 2020) and tested with linear trend of proportions to examine whether the new questionnaire was reliably incorporated into the process of appointment scheduling over time. We also examined differences in accessibility request estimates by geographic location; larger, hospital-based location (clinics at the Johns Hopkins Hospital in East Baltimore) vs smaller location (all satellite eye clinics). All analyses were conducted using Stata, version 14 (StataCorp). Statistical significance was set at *P* < .05, and 2-sided values are presented.

## Results

There were 269 856 patient encounters at the Wilmer eye clinics during the study period, of which 18 924 (7.0%) had missing data in the accessibility requests field. The first quarter (April to June 2019) had the greatest proportion of missing data on accessibility requests (17 449 of 68 889 [25.3%]), and the trend for missing data showed significant reductions in the subsequent second (870 of 67 694 [1.3%]), third (453 of 67 005 [0.7%]), and fourth (152 of 66 268 [0.2%]) quarters of the study period (*P* for trend <.001).

A total of 250 932 patient encounters with data on accessibility requests were included in this analysis. The patients had a mean (SD) age of 61.9 (20.6) years. Most were women (146 846 [58.5%]) and White individuals (162 720 [64.9%]). Of the total patients, 9.4% (n = 23 510) reported having at least 1 accessibility request. Compared with patient encounters not reporting an accessibility request, patients with encounters associated who had an accessibility request were older (mean [SD] age, 72.6 [18.0] vs 60.8 [20.5] years), more likely to be female (62.6% vs 58.1%), less likely to be Hispanic (2.9% vs 4.0%) and White individuals (59.7% vs 65.4%), more likely to be covered by any Medicare (rather than only private or other insurance) (69.6% vs 41.5%), and more likely to have vision impairment (41.3% vs 13.6%; *P* < .001 for all) (Table 1). In addition, they were less likely to be new patient encounters (19.2% vs 23.5%) and more likely to be follow-up appointments (71.3% vs 67.8%; *P* < .001).

**Table 1.** Patient Encounter Characteristics at the Johns Hopkins Wilmer Eye Institute From April 2019 to March 2020^a^

Characteristic	Patient encounters, No. (%)			*P* value
	Total (n = 250 932)	No accessibility requests (n = 227 422 [90.6%])	Accessibility request (n = 23 510 [9.4%])	
Age, mean (SD), y	61.9 (20.6)	60.8 (20.5)	72.6 (18.0)	<.001
Sex				
Female	146 846 (58.5)	132 123 (58.1)	14 722 (62.6)	<.001
Male	104 076 (41.5)	95 289 (41.9)	8787 (37.4)	
Hispanic ethnicity	9296 (3.7)	8642 (4.0)	654 (2.9)	<.001
Race				
Asian	13 198 (5.3)	12 663 (5.6)	535 (2.3)	<.001
Black	56 028 (22.3)	48 426 (21.3)	7602 (32.3)	
White	162 720 (64.9)	142 677 (65.4)	14 043 (59.7)	
Other^b^	18 986 (7.6)	17 656 (7.8)	1330 (5.7)	
Medicare	110 727 (44.1)	94 370 (41.5)	16 357 (69.6)	<.001
Medicare or Medicaid	112 014 (44.6)	95 267 (41.9)	16 747 (71.2)	<.001
Visit type				
New patient	57 974 (23.1)	53 463 (23.5)	4511 (19.2)	<.001
Follow-up	170 994 (68.1)	154 236 (67.8)	16 758 (71.3)	
Postoperative	19 625 (7.8)	17 691 (7.8)	1934 (8.2)	
Other^c^	2339 (0.9)	2032 (0.9)	307 (1.3)	
Vision impairment^d^	39 977 (16.2)	30 472 (13.6)	9505 (41.3)	<.001
Geographic location				
East Baltimore clinics	121 425 (48.4)	108 030 (47.5)	13 395 (52.5)	<.001
Satellite clinics	129 507 (51.6)	119 392 (57.0)	10 115 (43.0)	

^a^ Patients could have scheduled multiple clinic encounters during the study period.

^b^ Other race includes American Indian, Alaska Native, Native Hawaiian, Other Pacific Islander, 2 or more races, and unknown.

^c^ Other visit type includes visits documented as preoperative, procedures, surgical consultations, and testing, among others.

^d^ Based on better-eye visual acuity (best-available acuity used: either best-corrected presenting or pinhole based on availability) worse than 20/40.

The most commonly reported accessibility request was a mobility limitation (n = 18 857 [7.5%]), followed by sensory impairment (n = 2988 [1.2%]), other impairment (n = 1312 [0.5%]), and intellectual impairments (n = 353 [0.1%]) (Table 2). In an additional 2934 patient encounters during this study period, a second accessibility request was reported, most commonly a mobility request. There were no patient encounters associated with more than 2 requests. A slightly larger proportion of patient encounters had accessibility requests at the larger, hospital-based clinics than the smaller satellite clinics (Johns Hopkins Hospital in East Baltimore, 11.0% vs Wilmer satellite eye clinics, 9.4%; *P* < .001).

**Table 2.** Types of Accessibility Requests Reported in All Patient Encounters to the Johns Hopkins Wilmer Eye Institute From April 2019 to March 2020

Variable	No. (%)
Accessibility need	
Total No.	250 932
None	227 422 (90.6)
Mobility	18 857 (7.5)
Sensory	2988 (1.2)
Intellectual	353 (0.1)
Other	1312 (0.5)
Additional accessibility need^a^	
Total No.	2934
Mobility	2252 (76.8)
Sensory	434 (14.8)
Intellectual	37 (1.3)
Other	211 (7.2)

^a^ Additional accessibility requests reported among those reporting a primary accessibility request in all patient encounters.

Of the 250 932 patient encounters analyzed, 55 722 (22.2%) were unique patients and the remainder were repeated appointments. During this study period, these patients had a median (IQR) of 3 visits (2-5; range, 1-54). Table 3 examines patient characteristics for their first visit only during the study period, and the results were largely similar to findings from examination of all patient encounters, although only 5.2% of patients reported having at least 1 accessibility request during their first visit, and patients were younger.

**Table 3.** Patient Characteristics at First Visit to the Johns Hopkins Wilmer Eye Institute From April 2019 to March 2020

Characteristic	Patients, No. (%)			*P* value
	Total (n = 55 722)	No accessibility requests (n = 52 849 [94.8%])	Accessibility request (n = 2873 [5.2%])	
Age, mean (SD), y	54.4 (22.7)	53.8 (22.5)	65.8 (23.7)	<.001
Sex				
Female	32 763 (58.8)	31 043 (58.8)	1720 (59.9)	.24
Male		21 798 (41.2)	1153 (40.1)	
Hispanic	2312 (3.8)	2222 (4.5)	90 (3.3)	.005
Race				
Asian	3304 (5.9)	3223 (6.1)	81 (2.8)	<.001
Black	12 116 (21.7)	11 231 (21.3)	885 (30.8)	
White	35 538 (63.8)	33 823 (64.0)	1715 (59.7)	
Other^a^	4764 (8.6)	4572 (8.7)	192 (6.7)	
Medicare	17 440 (31.3)	15 700 (29.7)	1740 (60.6)	<.001
Medicare or Medicaid	17 835 (32.0)	15 992 (30.3)	1843 (64.2)	<.001
Visit type				
New	23 878 (42.9)	22 631 (42.8)	1247 (43.4)	.30
Follow-up	31 559 (56.6)	29 952 (56.7)	1607 (55.9)	
Postoperative	59 (0.1)	53 (0.1)	6 (0.2)	
Other^b^	226 (0.4)	213 (0.4)	13 (0.5)	
Vision impairment^c^	3169 (5.8)	2484 (4.8)	685 (25.6)	<.001
Geographic location				
East Baltimore clinics	21 528 (38.6)	20 024 (38.0)	1504 (52.4)	<.001
Satellite clinics	34 194 (61.4)	32 825 (62.1)	1369 (47.7)	

^a^ Other race includes American Indian, Alaska Native, Native Hawaiian, Other Pacific Islander, 2 or more races, and unknown.

^b^ Other visit type includes visits documented as preoperative, procedures, surgical consultations, and testing, among others.

^c^ Based on better-eye visual acuity (presenting or best-corrected based on availability) worse than 20/40.

## Discussion

We created and implemented an EHR-based questionnaire at an eye clinic to document patient accessibility requests. Over a 1-year period, almost 10% of patient encounters at an academic eyecare center included an accessibility request, most being mobility related, underscoring how common disability accessibility requests are in this setting. This questionnaire provides the infrastructure to collect accessibility requests, and such standardization of data collection can be scaled to other departments and clinical settings, with the potential to improve patient interaction and care.

To our knowledge, similar data are not available from eye clinics or other clinical settings in the US for us to compare accessibility request estimates across patient populations. However, national-level data show that up to 26% of the US population has at least 1 type of disability.^1^ A distinction to note is that this study did not identify all patients with disabilities, but instead the proportion of patients with disabilities who indicated a need for accommodations at an ophthalmic appointment. Not all patients with disabilities have accessibility requests. Some patients with disabilities may not need an accommodation, others may be familiar with the clinic setting and provide their own accommodation, and some patients with disabilities may be hesitant to make an accessibility request.

We found modest differences by geographic location of the clinics: a slightly larger proportion of patients reported accessibility requests at the larger, hospital-based eye clinics than the smaller satellite clinics (11% vs 9%, respectively), suggesting that either more patients with disabilities are using these clinics or that patients with disabilities are more likely to request assistance in more crowded and complex settings. For example, parking garages are situated farther away from the clinics in the downtown hospital setting, and the circuitous route to access the clinic likely compounded accessibility requests. We also noted that, compared with the 9.4% of all patient encounters where an accessibility request was made, only 5.2% of patients reported having an accessibility request during their first visit, suggesting that patients with disabilities were more likely to have multiple clinic visits and/or answer the EHR questionnaire while making an appointment. In addition, it must also be considered that first-visit patients are, on average, younger than returning patients.

Previous studies have found that people with disabilities experience more difficulty accessing health services and have lower rates of preventive screening compared with people without disabilities.^2,6,22^ For example, 22% of community residents with physical or sensory disabilities reported having difficulty accessing their health care clinician’s office, with up to 33% of people with severe disabilities reporting physical barriers.^23^ People with disabilities are also more likely to be unable to get or delay needed dental care or prescription medications in the past year than people without disabilities.^2,24^ In addition, women with physical, sensory, and cognitive disabilities have lower rates of mammography and cervical Papanicolaou testing than women with no disabilities.^25^

Reasons for health care disparities are likely complex, with interplay between social and environmental factors, such as disadvantages around education, income, and employment, in addition to competing needs from multiple health issues.^2,26,27^ Nevertheless, inaccessible health care equipment and facilities are likely still an important contributor to health care disparities that people with disabilities experience.^16,28^ Among its objectives for people with disabilities, Healthy People 2020 includes decreasing barriers in health care facilities. Furthermore, patient-centered care, respecting patient preferences, needs, and values, is listed as 1 of 6 key features of a high-quality health care system in the Institute of Medicine’s hallmark report.^2,29^ Therefore, ultimately, this is a health equity endeavor; to provide equitable access to navigate health care systems, we need to develop and implement better systems that measure and address accessibility requests for people with disabilities.

### Limitations

There are some study limitations to be considered. Any patients who requested appointments electronically (ie, via the Johns Hopkins MyChart, a web platform that connects patients to the health care team) did not encounter these accessibility request questions. In addition, only data in the EHR’s structured and newly piloted special needs fields (a term not determined by the investigators of this study) were examined. Therefore, any accessibility requests data documented in the unstructured fields or patients making appointments electronically were not captured. Second, “special needs” is not a term preferred by people with disabilities because it euphemistically stigmatizes that which is different, and future expansion of this EHR feature should use more inclusive language.^20,21^ Third, a substantial proportion (25.3%) of patient encounters were missing data on accessibility requests at the start of the project (April to June 2019) when the questionnaire was first implemented. However, the progressive reduction in missing data (to <1% missing data from July 2019 to March 2020) showed that staff use of the questionnaire improved following an initial transition period and that the questionnaire was reliably incorporated into the process of appointment scheduling. Fourth, study estimates of the proportions reporting accessibility requests are from a university-based eye clinic and may not be generalizable to other clinical or health care settings. Fifth, although we focused on patient encounter–level data in the primary analysis because disability is not a permanent fixture of an individual and can change over time, future research should examine changes and patterns in accommodation requests at a person level in robust analyses accounting for clustering at an encounter level. In addition, the accessibility requests assessed with this questionnaire in fact measured a mix of durable medical equipment (eg, motorized scooter or walker), functional impairments (eg, hearing impaired or visually impaired), and diagnoses (eg, intellectual disability). Future research should restructure queries around actionable accommodation needs, such as the need for American Sign Language interpretation instead of documenting hearing impairment. Such information will be useful for the clinic staff to arrange interpreter services in advance of appointments to help patients fully participate in their health care appointments.

Despite these limitations, this EHR-based questionnaire has the potential to improve accessibility of health care interactions for patients with disabilities. Although individuals’ accessibility request information was collected in this study, and wheelchairs, sign language interpreting, and other services listed in the Figure are available on request at the Wilmer Eye Institute, these requests might not have been fully accommodated. The next phase of this project is examining what proportion of accessibility requests are being met and how. In addition, although there are improvements in design and application to be made to this questionnaire based on findings from this pilot, it is a scalable design. There are opportunities to collect disability and accommodations data in all EHR systems, and at different phases of patient interactions. An important future step will be to work directly with developers of EHR software to ensure that accommodation request information is collected for all patients scheduling appointments.

## Conclusions

This EHR-based system provides a standardized method for collecting important accessibility requests for patients with disabilities at academic eye centers. This novel approach has the potential to be extended to other health care settings beyond ophthalmology. Future research is needed to assess whether accessibility requests are being met and the effectiveness of the questionnaire in improving health care accessibility, interactions, and outcomes for people with disabilities.

## Notes

Supplement. **eAppendix.** Survey questionnaire used by the call center and front-desk staff to enquire patients for accessibility requests
