# Supplementary material for: Identifying Accessibility Requests for Patients With Disabilities Through an Electronic Health Record–Based Questionnaire
Source: JAMA Netw Open. 2022 Apr 8;5(4):e226555. doi: 10.1001/jamanetworkopen.2022.6555 (PMC8994132; doi:10.1001/jamanetworkopen.2022.6555)
Supplement: Supplement. — eAppendix. Survey questionnaire used by the call center and front-desk staff to enquire patients for accessibility requests [file jamanetwopen-e226555-s001.pdf]

# Supplementary Online Content

Varadaraj V, Guo X, Reed NS, et al. Identifying accessibility requests for patients with disabilities through an electronic health record–based questionnaire. *JAMA Netw Open*. 2022;5(4):e226555. doi:10.1001/jamanetworkopen.2022.6555

**eAppendix.** Survey questionnaire used by the call center and front-desk staff to enquire patients for accessibility requests

This supplementary material has been provided by the authors to give readers additional information about their work.

© 2022 Varadaraj V et al. *JAMA Network Open*.

**eAppendix.** Survey questionnaire used by the call center and front-desk staff to enquire patients for accessibility requests

- If the Special Needs field is already populated, confirm with the patient whether the current accessibility requests on file are accurate for the next visit

- If the Special Needs field is blank, ask: "Do you have any accessibility requests for this visit?" If yes,
  - "Will you need any assistance traveling from our parking garage to the Wilmer Building?" If yes,
    - "Would you like me to request assistance from Patient Transportation Service?" If yes,
      - "Do you need any special equipment besides a standard sized wheelchair?"
  - "Do you require assistance with seeing, reading, or completing forms?" If yes,
    - "Will you have someone accompanying you for your appointment?" If no,
      - "I will make a note in your appointment slot, and we can provide you assistance when you arrive."
  - "Do you have difficulty with mobility?" If yes,
    - "Do you use a mobility aid such as a wheelchair or a walker?" If yes,
      - "Please describe the equipment"
    - "Are you able to stand independently?"
  - "Will you be accompanied by a service animal?" If yes,
    - "Is the animal required because of a disability?"
    - "What task/tasks is/are the animal trained to perform?"
    - "What kind of animal is it?"
  - For patients who have intellectual disabilities
    - "Can you (the patient) understand simple questions and instructions?"
    - "Can you (the patient) understand health care questions and instructions?"

    If no, try to get details of the limits of the patient's understanding

© 2022 Varadaraj V et al. *JAMA Network Open*.
